# Massive Transformation in Titanium-Silver Alloys and Its Effect on Their Mechanical Properties and Corrosion Behavior

**DOI:** 10.3390/ma7096194

**Published:** 2014-08-29

**Authors:** Mi-Kyung Han, Moon-Jin Hwang, Dae-Hee Won, Yang-Soo Kim, Ho-Jun Song, Yeong-Joon Park

**Affiliations:** 1 Department of Dental Materials and Medical Research Center for Biomineralization Disorders, School of Dentistry, Chonnam National University, Gwangju 500-757, Korea; E-Mails: mikihan@jun.ac.kr (M.-K.H.); mjhwang@jnu.ac.kr (M.-J.H.); songhj@jnu.ac.kr (H.-J.S.); 2 Industry-Academic Cooperation Foundation, Wonkwang University, Jeonbuk 570-749, Korea; E-Mail: oneday@wku.ac.kr; 3 Sunchon Center, Korea Basic Science Institute, Sunchon 540-950, Korea; E-Mail: kimyangsoo@kbsi.re.kr

**Keywords:** Ti–Ag alloys, dental materials, corrosion resistance, mechanical properties, microstructure

## Abstract

In order to investigate the relationship between phase/microstructure and various properties of Ti–*x*Ag alloys, a series of Ti–*x*Ag alloys with Ag contents ranging from 5 to 20 wt% were prepared. The microstructures were characterized using X-ray diffractometry (XRD), optical microscopy (OM), scanning electron microscopy (SEM) and transmission electron microscopy (TEM). All of the Ti–*x*Ag alloys showed a massive transformation from the β-Ti to α_m_ phase, which has a different crystal structure from that of the matrix phase, but it has the same composition as the matrix α-Ti phase. As a result of solid-solution strengthening of α-Ti and massive transformation phase, the Ti–*x*Ag showed better mechanical properties than the commercially pure titanium (cp-Ti). Electrochemical results showed that the Ti–*x*Ag alloys exhibited improved corrosion resistance and oxidation resistance than cp-Ti.

## 1. Introduction

Ti–based alloys have been widely used in fabrication of dental and orthopedic implants due to their favorable mechanical properties, including high strength-to-density ratio, good corrosion stability and high biocompatibility [1,2]. However, it is necessary to improve the mechanical properties of Ti for practical application in dentistry.

Recently, binary Ti–Ag alloys have been developed for use in dental applications, due to their excellent corrosion resistance, improved mechanical properties and biocompatibility compared to commercially pure titanium (cp-Ti) [3,4,5,6,7]. In addition, the Ti–Ag alloys have a low ion-dissolution rate in NaCl solution [8]. Another study found that Ag-coated Ti has an improved antibacterial effect, as well as good cell compatibility [9,10]. Due to the fact that intermetallic compounds, such as Ti_2_Ag and TiAg, deteriorate the corrosion resistance of Ti, the amount of Ag added to the Ti should be less than 25 wt% for use as dental materials [8]. The cytotoxicity tests performed by Zhang *et al.* show that Ti–Ag alloys exhibited similar cell viability to that of cp-Ti [11]. Other than this, there has been little experimental investigation about the relationship between phases/microstructure and the mechanical properties of Ti–Ag alloys. Therefore, it is important to pursue a comprehensive structural analysis of Ti–Ag alloys, which is necessary for developing new Ti–based alloys with desired properties.

Under certain conditions, several metallic alloys exhibit a composition-invariant transformation, which is termed massive transformation. Massive β → α_m_ transformation has been observed in Ti–*x*Ag alloys containing Ag ≤ 26 wt% [12]. In the present study, Ti–Ag binary alloys with the addition of 5, 10, 15 and 20 wt% Ag were fabricated by arc melting. Massive transformation in the Ti–Ag alloys was observed using X-ray diffractometry (XRD), optical microscopy (OM), scanning electron microscopy (SEM) and transmission electron microscopy (TEM). In order to investigate the effects of massive transformation in Ti–Ag alloys, the following analyses were carried out. Microhardness and elastic modulus were measured using a Vickers microhardness tester and a nanoindenter. Phase transformation and oxidation behavior were investigated by simultaneous differential scanning calometer (DSC) analysis and thermogravimetric analysis (TGA). Corrosion stability was evaluated through potenti odynamic polarization and galvanic corrosion tests in 0.9% NaCl solution at 37 ± 1 °C. In this work, “Ti–*x*Ag” will henceforth stand for “Ti–*x* wt% Ag” in the text.

## 2. Results and Discussion

### 2.1. Phase and Microstructure

**Figure 1:**
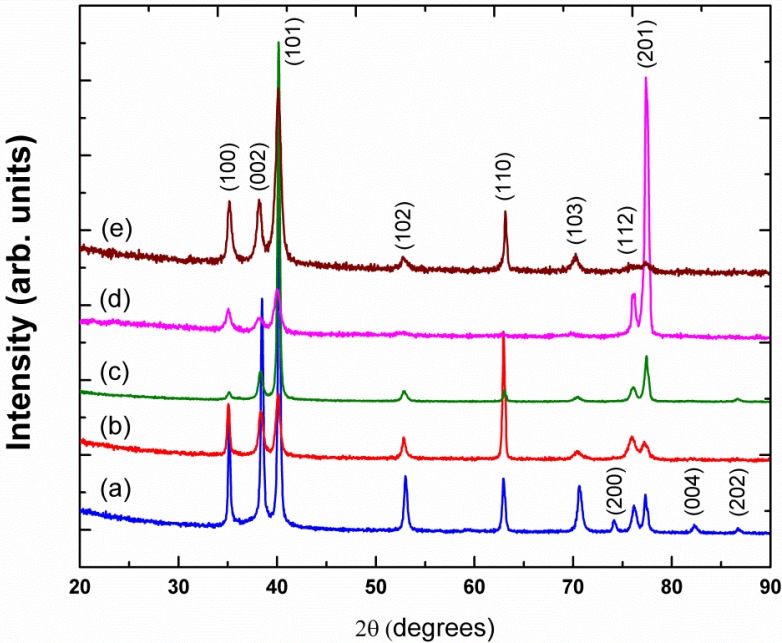
X-ray diffractometry (XRD) patterns of the cast commercially pure titanium (cp-Ti) and the series of binary Ti–*x*Ag (Ti–*x* wt% Ag) alloys. (**a**) cp-Ti; (**b**) Ti–5Ag; (**c**) Ti–10Ag; (**d**) Ti–15Ag; and (**e**) Ti–20Ag.

The X-ray diffraction patterns as a function of *x* for the Ti–*x*Ag (*x* = 5, 10, 15 and 20 wt%) samples are shown in Figure 1 and compared with the XRD pattern of cp-Ti. The patterns of Ti–*x*Ag alloys could be indexed in the hexagonal α-Ti type structure, the space group of *P*6_3_/*mmc*, with no indication of the existence of a secondary phase. This result was consistent with the previous analysis of phases in the Ti–Ag system [13,14]. With the exception of dilute Ti–5Ag and Ti–10Ag alloys, binary Ti–Ag alloys commonly tend to precipitate intermetallic compounds Ti_2_Ag and TiAg when equilibrium solidification is considered. In light of this, at the eutectoid temperature, microstructure arrays constituted of the “α-Ti + intermetallic Ti_2_Ag” phase form from the beta phase. In this study, however, owing to non-equilibrium solidification, no such intermetallic compounds were detected, in agreement with the results previously reported by Takahashi *et al.* [15].

**Figure 2:**
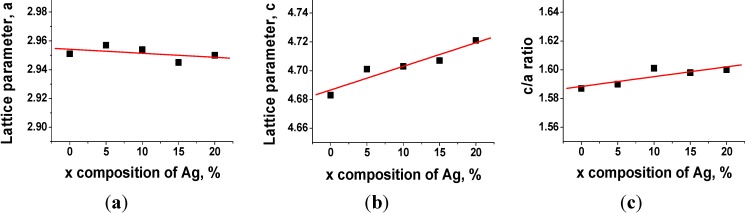
(**a**,**b**) Lattice parameters (*a* and *c*) of cp-Ti and as-cast Ti–*x*Ag alloys and (**c**) the variation in the ratio (*c*/*a*) of lattice parameters.

Rietveld refinements were performed in the present work to investigate the effect of Ag on the lattice parameters. The lattice parameter variations of Ti–*x*Ag alloys as a function of Ag content are presented in Figure 2. Due to the similar atomic radius of Ag (~1.44 Å) and Ti (~1.47 Å), the alloying between Ti and Ag occurred as a solid solution. The addition of Ag atoms caused an increase in the lattice parameter *c*, whereas the lattice parameter *a* remained constant. As a result, the *c*/*a* ratio increased slightly as the content of Ag increased. Unit cell constants of cp-Ti were *a* = 2.951(1) Å and *c* = 4.683(1) Å (*c*/*a* ratio = 1.581), and they corresponded well with those in the literature (JCPDS card No. 44-1294), whereas the unit cell parameters of Ti–20Ag were *a* = 2.950(1) Å and *c* = 4.721(1) Å (*c*/*a* ratio = 1.600). The trends of lattice parameters based on the Ag content were in agreement with the result obtained in a previous study [16].

**Figure 3:**
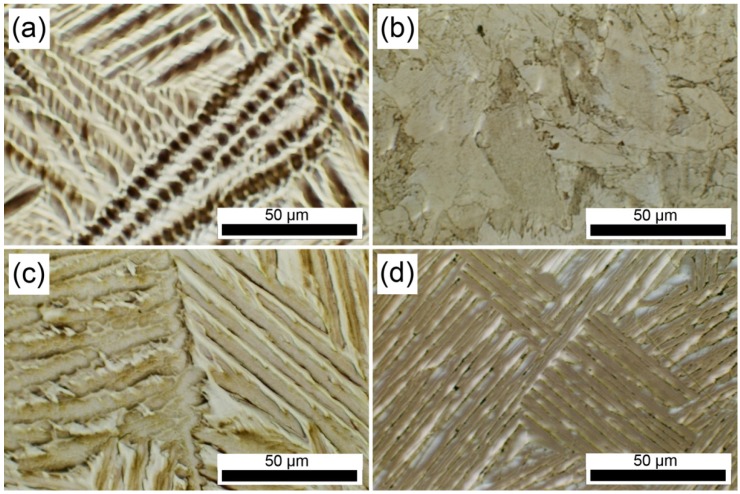
Optical micrographs of Ti–*x*Ag alloys; (**a**) Ti–5Ag; (**b**) Ti–10Ag; (**c**) Ti–15Ag; and (**d**) Ti–20Ag.

**Figure 4:**
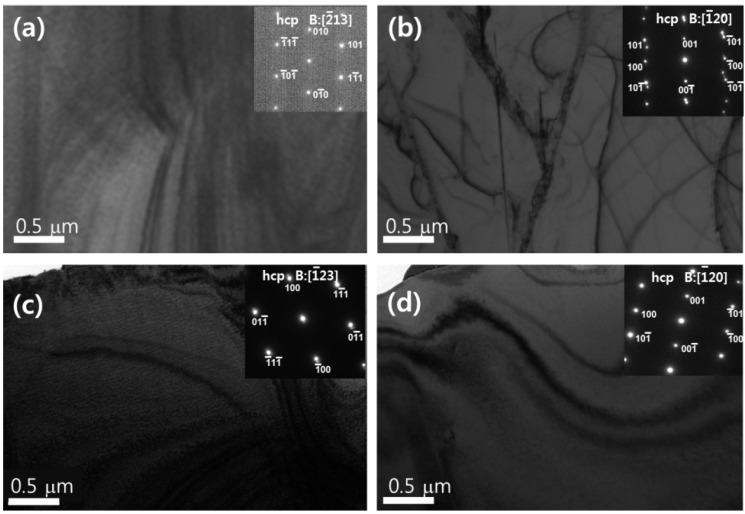
TEM micrographs of Ti–*x*Ag alloys; (**a**) Ti–5Ag; (**b**) Ti–10Ag; (**c**) Ti–15Ag; and (**d**) Ti–20Ag.

Massive β → α_m_ transformation is known to occur in the Ti–*x*Ag alloys containing Ag ≤ 26 wt% [12]. The product phase has a different crystal structure from that of the matrix phase, but it has the same composition as the matrix phase. Therefore, massive transformation is composition invariant, but it shows an incoherent interface. Figure 3 and Figure 4 show the OM and TEM images of Ti–*x*Ag alloys with different Ag contents (5, 10, 15 and 20 wt%). In Ti–10Ag, the grains of Ti–5Ag changed to an equiaxed structure with irregular grain boundaries, as shown in Figure 3a,b. Acicular structures were observed in Ti–Ag alloys having Ag contents above 10 wt%, and these structures became smaller with increasing Ag content, as shown in Figure 3c,d. The matrix was composed of the α-Ti phase, as indicated by the selected area energy diffraction (SAED) pattern (inset in Figure 4).

**Figure 5:**
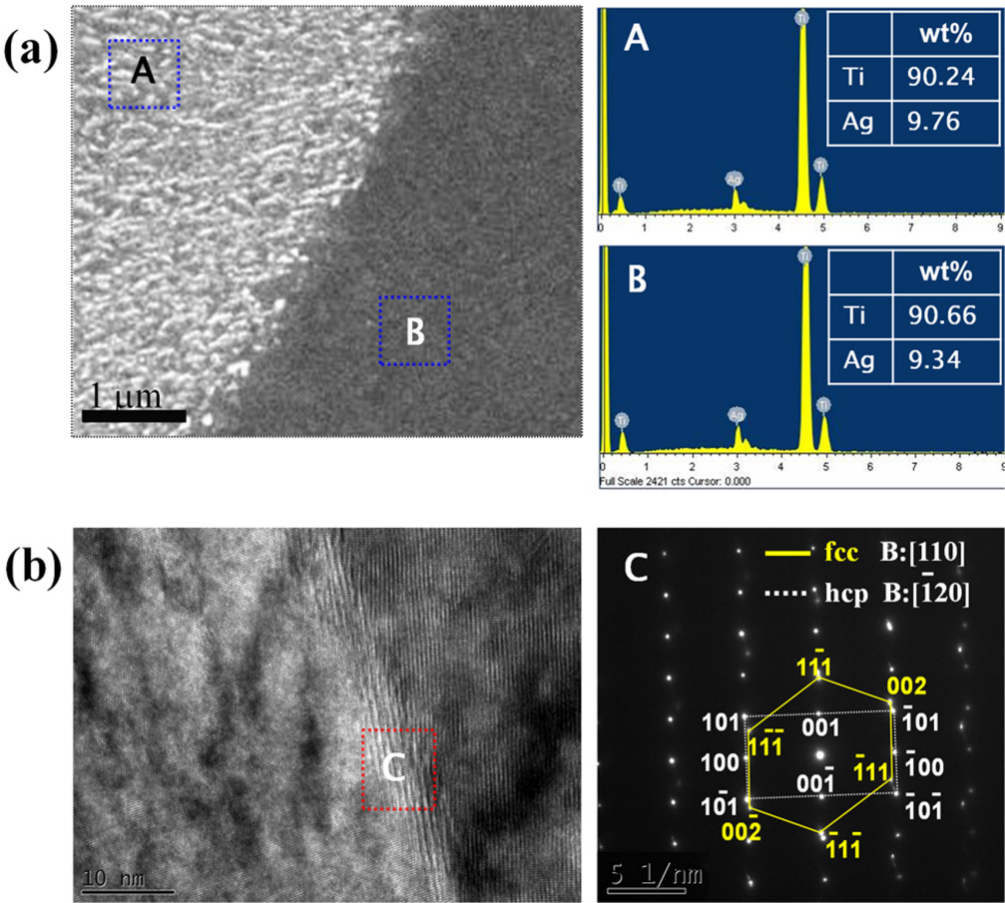
(**a**) SEM micrograph with quantitative EDX analysis of Ti–10Ag alloy; and (**b**) HR-TEM micrograph with the SAED pattern of the Ti–10Ag alloy.

Confirmation of this massive transformation was provided by qualitative energy dispersive X-ray (EDX) analysis of Ti–10Ag alloys. SEM analysis of polished surfaces showed two different morphologies, which are marked as A and B in Figure 5a. The EDX analysis revealed that, within experimental limits, the composition of both morphologies was identical. Therefore, the observed transformation was composition invariant. Massive transformation was reconfirmed through observation of the microstructure using high-resolution transmission electron microscopy (HR-TEM). Figure 5b shows the representative TEM image for the Ti–10Ag alloy. HR-TEM showed that the phase interface was incoherent. The SAED patterns of the Ti–10Ag alloy consisted of the characteristic hexagonal close packed (hcp) Ti phase of the matrix, showing perfect atomic arrangement and the sub-microstructure of the face centered cubic (fcc) Ti phase. The corresponding SAED patterns of the sub-microstructure could be indexed in terms of the [110] zone axis of the cubic structure of Ti.

**Figure 6:**
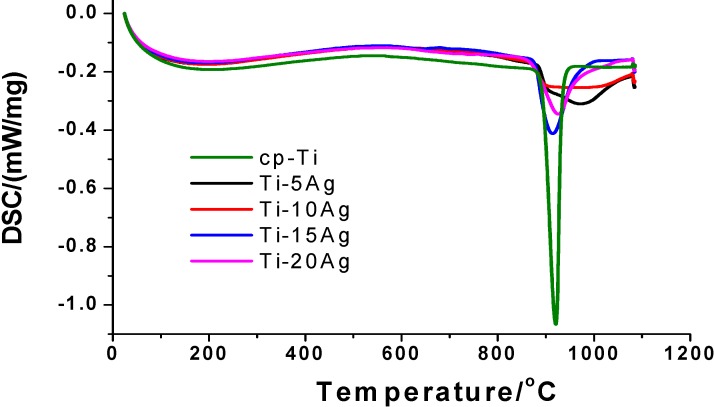
Differential scanning calorimetry (DSC) curves of the cast cp-Ti and series of binary Ti–*x*Ag alloys.

Differential scanning calorimetry (DSC) analysis was performed to detect any abnormal thermal effects from room temperature to 1000 °C, which could be indicative of the presence of a free segregation of Ag or intermetallic precipitates. As shown in Figure 6, very smooth temperature-dependent traces were found at about 150 °C for all of the Ti–*x*Ag alloys with Ag content up to 20 wt%. This peak resulted from the stabilization of the thermally unstable structure, such as grain boundary relaxation or grain boundary reordering. The second endothermic peak at about 900 °C resulted from the martensitic transformation of α-Ti to β-Ti. Other than these two peaks, no extraneous exothermic or endothermic peaks were detected. Thus, based on the XRD, SEM, TEM and DSC results, we could conclude that the nominal 20 wt% content of Ag used in this work did not exceed the solubility limit of Ag in the Ti alloys.

**Figure 7:**
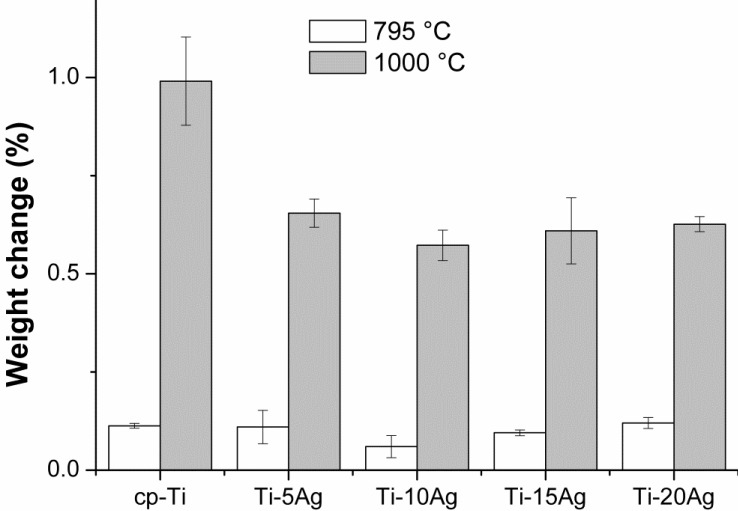
Thermogravimetric analysis (TGA) of cp-Ti and Ti–*x*Ag alloys showing various degrees of weight gain (%) by heating in air up to 795 °C and 1000 °C.

The oxidation behavior of Ti–*x*Ag alloys was assessed using thermogravimetric analysis (TGA). Figure 7 shows the result of the TGA experiment when the cp-Ti and Ti–*x*Ag alloys were heated up to 795 °C and 1000 °C at a heating rate of 10 °C/min in air. Each sample was oxidized, and the weight gain in the oxidized samples was compared with the weight of the non-oxidized samples. All of the samples showed a single parabolic increment in mass during oxidation. The change in mass was not observed between room temperature to 600 °C in all of the Ti–*x*Ag alloys, which was indicative of the oxidation resistance. At temperatures higher than 600 °C, the mass of the Ti–*x*Ag alloys increased exponentially. The weight change was increased with an increase in the oxidation temperature. The final mass change in the Ti–*x*Ag alloys was significantly less than that in the cp-Ti, indicating that the addition of Ag to cp-Ti could restrain the oxidation rate of the alloy, and Ti–*x*Ag alloys had a high oxidation protection ability. The weight gain was decreased by increasing the Ag content up to 15 wt%, and then, it was increased by increasing the Ag content further. Therefore, Ti–*x*Ag alloys had a higher oxidation protection ability, and the addition of Ag to cp-Ti could restrain the oxidation rate of the alloy.

### 2.2. Mechanical Properties

The Vickers hardness means and elastic modulus of each Ti–*x*Ag alloy are shown in Table 1, and these values are compared to those of cp-Ti. The Ag element could effectively increase the microhardness values of cp-Ti (165 Vickers Hardness (VHN)), which could be explained by solid-solution strengthening of the α-phase, as suggested in a previous study [17,18]. Among the Ti–*x*Ag alloys, Ti–10Ag exhibited the highest hardness value of 501 VHN. This increase in hardness was probably caused by the massive transformation of the α phases along with solid-solution strengthening. The elastic modulus of cp-Ti was 132 GPa. The elastic modulus of Ti–5Ag was slightly higher than that of cp-Ti, whereas the elastic moduli of the other Ti–*x*Ag alloys (*x* = 10, 15 and 20) were lower (*p* < 0.05) than that of the cp-Ti. The lowering of the elastic modulus by alloying Ti with Ag atoms was attributed to the imperfect α phases during massive transformation [13]. The elastic modulus values for the Ti–*x*Ag (*x* = 10, 15 and 20 wt%) alloys were in the range of 122–130 GPa, which is comparable to the previously reported values [19].

**Table 1:** Vickers hardness and elastic modulus values of Ti–*x*Ag alloys compared to cp-Ti (*n* = 5).

Alloy Code	Vickers Hardness (VHN)	Elastic Modulus (GPa)
cp-Ti	165.0 (2.6) ^a,^*	132.4 (12.2) ^b,c,^*
Ti–5Ag	251.7 (1.2) ^b^	140.4 (4.9) ^c^
Ti–10 Ag	501.0 (17.3) ^c^	126.4 (8.5) ^a,b^
Ti–15Ag	275.3 (11.0) ^b^	129.6 (7.0) ^a,b^
Ti–20 Ag	485.0 (68.4) ^c^	122.7 (4.6) ^a^

* Within the same column, mean values with the same superscript letter were not statistically different at 5% (*p* > 0.05) by the Duncan’s multiple range test.

### 2.3. Corrosion Behavior

**Figure 8:**
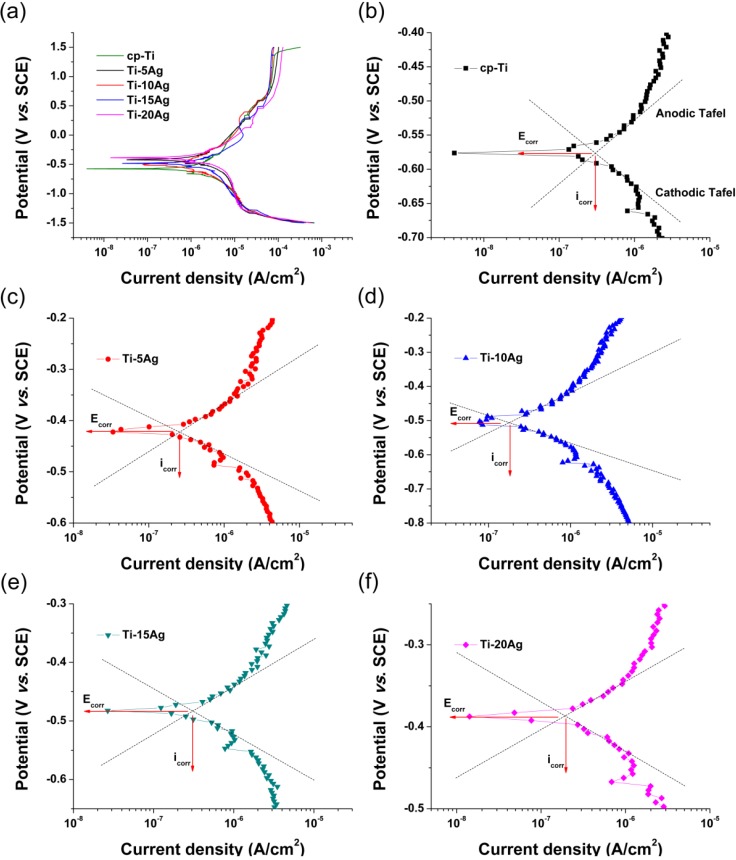
Representative potentiodynamic polarization curves for the cp-Ti and Ti–*x*Ag alloys. SCE, saturated calomel electrode.

Potentiodynamic polarization and the galvanic couple technique were used to investigate the effect of Ag content on corrosion resistance. The potentiodynamic polarization curves of the cp-Ti and Ti–*x*Ag alloys were recorded at a sweep rate of 5 mV/s in the potential range of −1.5 to 1.5 V in deaerated 0.9% NaCl solution (pH = 7.02), and the results are shown in Figure 8a. The initial passive potential of Ti–*x*Ag alloys showed a shift in the positive direction as compared to that of cp-Ti, which indicated a remarkable enhancement in the corrosion resistance by Ag addition. The initial passive current densities (~12.8 μA/cm^2^) of Ti–10Ag and Ti–15Ag were observed to be smaller than that (21.70 μA/cm^2^) of cp-Ti. The increase in the initial passive current density was observed for Ti–5Ag (21.28 μA/cm^2^) and Ti–20Ag (25.83 μA/cm^2^). Figure 8b–f show the Tafel plots obtained from the representative potentiodynamic polarization curves of the cp-Ti and Ti–*x*Ag alloys (Figure 8a).

Using the Tafel extrapolation method, we calculated the corrosion potential (*E*_corr_) and corrosion current density (*i*_corr_) of cp-Ti and Ti–*x*Ag alloys using both anodic and cathodic branches of the potentiodynamic polarization curves, and they are listed in Table 2. The average corrosion potential and current density values of all of the investigated Ti–*x*Ag alloys were respectively higher and lower than those of cp-Ti. Although no statistically significant difference was observed in the *i*_corr_ values among the tested cp-Ti and Ti–*x*Ag alloy samples, Ti–10Ag showed a slightly higher *i*_corr_ value. It is presumed that the decreased *E*_corr_ and increased *i*_corr_ values of Ti–10Ag can be attributed to the irregular boundaries of its equiaxed grains. With the exception of Ti–10Ag, the *E*_corr_ values for all alloys increased with increasing silver content, with microstructural changes to acicular forms and smaller phases. These results demonstrated that alloying Ti with Ag increased the corrosion resistance, which was probably caused by the accumulation of noble Ag atoms on the surface of Ti–Ag alloys due to the loss of Ti during the corrosion process [4,20]. Zhang *et al.* [11] reported that Ti–10Ag has a corrosion resistance expressed as a function of electrochemical impedance behavior lower than those of both Ti–5Ag and Ti–20Ag alloys. It seems that the eutectoid reaction is intimately associated with the decrease in the corrosion resistance of Ti–10Ag compared with the other two examined Ti–Ag alloys. It is well-known that the microstructure array resulting from the eutectoid reaction plays an important role in determining the electrochemical behavior, as previously reported for a number of as-cast Ti–based alloys [21,22] and various other non-ferrous alloys [23,24].

**Table 2:** Corrosion potential (*E*_corr_) and corrosion current density (*i*_corr_) of cp-Ti and Ti–*x*Ag alloys (*n* = 3).

Alloy Code	*E*_corr_ (mV)	*i*_corr_ (μA/cm^2^)
cp-Ti	−550.33 (43.94) ^a,^*	0.287 (0.046) ^a,^*
Ti–5Ag	−471.07 (101.37) ^a,b^	0.209 (0.072) ^a^
Ti–10Ag	−500.20 (31.82) ^a,b^	0.231 (0.057) ^a^
Ti–15Ag	−461.33 (19.98) ^a,b^	0.181 (0.120) ^a^
Ti–20Ag	−416.98 (58.72) ^b^	0.221 (0.078) ^a^

* Within the same column, mean values with the same superscript letter were not statistically different at 5% (*p* > 0.05) by the Duncan’s multiple range test.

**Figure 9:**
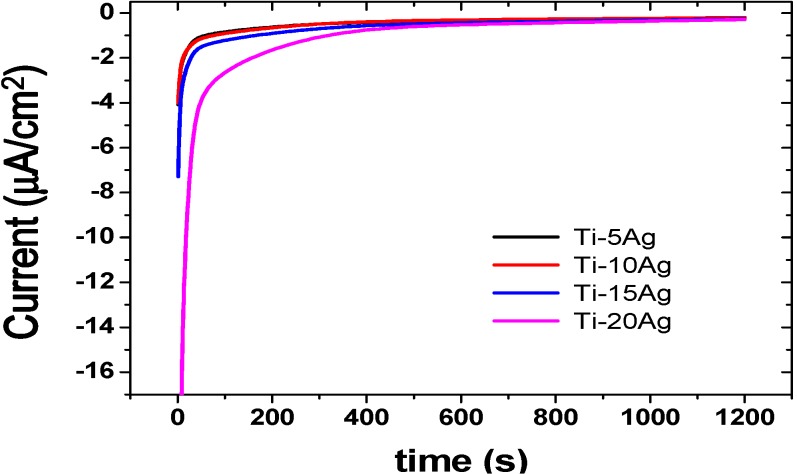
Mean values of galvanic currents *vs.* time of the couplings of cp-Ti/Ti–*x*Ag alloys.

Mean values of galvanic currents *versus* time of the couplings of cp-Ti/Ti–*x*Ag alloys are shown in Figure 9. All of the Ti–*x*Ag alloys apparently behaved in a similar manner. Current values initially exhibited a rapid increase and then leveled off. This general behavior might be explained by the reduction in the active area due to the growth of a passive film on the surface of cp-Ti. A steady-state current value was attained more rapidly for Ti–5Ag and Ti–10Ag compared to cp-Ti, indicating that the passive film grew more rapidly on cp-Ti. The time required for the Ti–*x*Ag alloys to attain a constant current was increased as the amount of Ag increased.

## 3. Experimental Section

### 3.1. Material Preparation

A commercially-available cp-Ti (ASTM Grade II, Daito Steel Co. Ltd., Tokyo, Japan) was used as the control titanium material. Experimental Ti–Ag alloys (5, 10, 15 and 20 mass% Ag) were prepared by arc-melting the stoichiometric quantities of the elements on a water-cooled copper hearth using a tungsten electrode under a high-purity argon atmosphere. The starting materials (Ti sponge, Alfa Aesar, Ward Hill, MA, USA, 99.9%; Ag ingot, LS-Nikko, Seoul, Korea, 99.95%) were used without purification. During the arc-melting procedure, a titanium getter was heated prior to melting the reactant mixture to further purify the argon atmosphere. The samples were remelted seven times to promote sample homogeneity. Subsequently, the samples were heat treated using a tube furnace under argon atmosphere for 4 h at temperatures 150 °C below the respective solidus temperatures followed by cooling down to 600 °C in a furnace at a rate of 10 °C/min and air-cooling to room temperature. These heat treatment conditions were chosen in accordance with the binary Ti–Ag phase diagrams [16].

### 3.2. Material Characterization

Phase analysis and structural characterization were performed by X-ray diffraction (XRD). The XRD diffraction patterns were collected for the bulk sample using a X’Pert PRO Multi–Purpose X-Ray Diffractometer (40 kV and 40 mA, PANalytical B.V., Almelo, The Netherlands) with Cu K_α_ (λ = 1.54056 Å). Intensities of the XRD were obtained in the 2θ range between 20° and 90° with a step size of 0.02°/3 s. The lattice parameters were obtained by least squares refinement of data in the 2θ range of 20°–90° with the aid of a Rietveld refinement program [25]. The microstructure of samples was examined using optical/metallurgical microscopy (Epiphot FX-35WA, Nikon, Tokyo, Japan), scanning electron microscopy (SEM; Hitachi, S-3000N, Tokyo, Japan.), high-resolution transmission electron microscopy (HR-TEM; Philips, Technai-F20, Amsterdam, The Netherlands), selected area energy diffraction (SAED) and energy dispersive X-ray analysis (EDX; EMAX, Horiba, Kyoto, Japan). Compositions of the cast Ti–*x*Ag alloys were determined using a scanning electron microscopy/energy dispersive spectrometry (SEM/EDS). Data collection was performed with a 20-kV accelerating voltage and 15% of mean dead time. The acquisition rate was 6 kcps, and the magnification was ×200. To carry out the quantitative analyses, pure Ag (99.95%) and Ti (99.99%) elements were used as standards (Taylor standard block, C60-001, CM Taylor Company, Amelia, VA, USA).

The phase transformation in Ti–*x*Ag alloys was investigated by heating approximately 200 mg of the sample to 1000 °C at a heating rate of 20 °C/min using a differential scanning calorimeter (DSC, DSC 404 C, Netzsch, Selb, Germany). The oxidation behavior of cp-Ti with different Ag contents was tested with TGA (SDTA 851e, Mettler-Toledo, Columbus, OH, USA), which measures the change in mass due to oxidation. The samples measuring 4.5 mm × 4.2 mm × 14.0 mm in size were heated to 795 °C or 1000 °C at a heating rate of 10 °C/min with an air flowing rate of 50 mL/min. Duplicate samples were tested per each test group.

### 3.3. Measurement of Mechanical Properties

For the measurement of mechanical properties, samples embedded in epoxy resin were cut and polished into disks of about a 1.2-mm thickness with successively finer SiC papers up to #2000 grit and then ultrasonically cleansed in distilled water. Subsequently, the polished samples were etched with Keller’s solution (distilled water: 65%; HNO_3_: 32%; HCl: 40%; HF = 95:2.5:1.5:0.5). The microhardness of polished alloys was measured using a Vickers microhardness tester (Zwick, Postfach4350, Ulm, Germany) with a load of 500 g for 30 s. Elastic modulus measurement was performed using Nanoindenter (MTS Company, Dubuque, IA, USA) in a continuous stiffness measurement mode with the Berkovich-type indenter. The indentations were made using a constant nominal strain rate of 5 × 10^−2^ s^−1^. The maximum indentation depth was 2 μm. A Poisson’s ratio of 0.35 was used to calculate the elastic modulus.

### 3.4. Electrochemical Analysis

The potentiodynamic anodic polarization test was conducted at a scan rate of 5 mV/s from −1.5 to +1.5 V (*vs.* SCE (saturated calomel electrode)) using a potentiostat (WAT 100, WonA Tech Co., Ltd., Seoul, Korea) in 0.9% NaCl solution at 37 ŷ 1 °C. At least three samples were tested to confirm the repetition of the experimental results. The surface of the sample with an approximately 10-mm diameter was mechanically polished with SiC paper up to 2000 grit. The electrochemical measurements were recorded using the three electrode technique consisting of the working electrode (test samples), the counter electrode (high density carbon) and the reference electrode (saturated calomel electrode). Before measurements, argon gas was bubbled through the electrolyte at 150 mL/min for more than 20 min to eliminate the residual oxygen in the electrolyte. Fresh electrolyte was used for each measurement. The exposed surface area of samples in the electrolyte was 0.283 cm^2^. Potentiodynamic anodic polarization measurements were carried out after an immersion period of 1 h at open circuit potential. The potentiodynamic polarization curves were plotted using an automatic data acquisition system. Corrosion potential and current density were estimated by the Tafel plots using both anodic and cathodic branches. The galvanic current densities of various Ti–*x*Ag/cp-Ti galvanic pairs were measured over a 20-min period by using a potentiostat/galvanostat at ambient conditions (ZIVE SP2, WonA Tech Co., Ltd., Seoul, Korea). The experimental setup for electrochemical measurements consisted of a three-electrode cell with the sample as a working electrode with an exposed area of 0.785 cm^2^, a saturated calomel electrode (SCE) as a reference electrode and cp-Ti as the counter electrode.

### 3.5. Statistical Analysis

Version 19.0 of the statistical software, SPSS (SPSS, Inc., Chicago, IL, USA), was used to analyze the data by means of the Kruskal–Wallis one-way analysis of variance and Duncan’s multiple range test with α = 0.05. Data were expressed as the mean ± standard deviation (SD) for each of the tests.

## 4. Conclusions

This study investigated the influence of Ag addition on the microstructure, mechanical properties and corrosion behavior of commercially pure titanium (cp-Ti). The Ti–*x*Ag alloys exhibited α-Ti structure at a silver content below 20 wt%. Based on the EDX and HR-TEM analysis, all of the Ti–*x*Ag alloys showed a massive transformation from the β-Ti to α_m_ phase, which has a different crystal structure from that of the matrix phase, but it has the same composition as the matrix α-Ti phase. As a result of solid-solution strengthening of α-Ti and massive transformation phase, the Ti–*x*Ag exhibited higher hardness and better oxidation protection ability than the cp-Ti. Electrochemical results showed that the Ti–*x*Ag alloys exhibited improved corrosion resistance compared to cp-Ti. After considering the mechanical properties and corrosion behavior of Ti–*x*Ag alloys, Ti alloys with Ag addition can be considered as good candidates for dental casting alloys.
